# The impact of COVID-19 on the male reproductive system

**DOI:** 10.5935/1518-0557.20240048

**Published:** 2024

**Authors:** Abzal Kystaubayev, Askhan Abzalbekov, Bakyt Ramazanova, Vyacheslav Lokshin, Muhammed Iskakov

**Affiliations:** 1 Asfendiyarov Kazakh National Medical University, Department of Microbiology, Virology. Almaty, Republic of Kazakhstan; 2 Kazakh-Russian Medical University, Department of Urology and Andrology. Almaty, Republic of Kazakhstan; 3 International Academy of Fertility Science. Almaty, Republic of Kazakhstan; 4 Asfendiyarov Kazakh National Medical University, Department of Health Politics and Management. Almaty, Republic of Kazakhstan

**Keywords:** SARS-CoV-2, spermatogenesis, fertility, ejaculation, sexual health

## Abstract

**Objective:**

The relevance of the study is determined by the deepening understanding of the global consequences of the coronavirus pandemic, which affect not only lung health but also a wide range of other body systems. In light of new data on the long-term effects of coronavirus infection, this study is highly significant. The purpose of this study is to investigate the impact of coronavirus infection on the male reproductive system and assess its potential influence on male fertility to refine the mechanisms of damage and provide recommendations for medical care.

**Methods:**

The study utilised a combination of methods, including a meta-analysis of medical organisation databases, analysis of clinical cases, representative sample method, and quantitative survey method. These approaches allowed for a comprehensive and multifaceted view of the problem.

**Results:**

The samples of sperm showed a noticeable decrease in progressive motility, sperm concentration, and volume, especially in patients with moderate and severe symptoms of COVID-19, whereas patients with mild symptoms only experienced a decrease in progressive motility and overall sperm motility. The survey identified symptoms of male reproductive system dysfunction after recovering from COVID-19. Predominant symptoms included decreased libido (15%), impotence (13%), and infections of the genital organs (12%). Most surveyed men lacked sufficient awareness of other aspects of male reproductive health, including infections, genetic defects, chronic diseases, and available medical services.

**Conclusions:**

As a result of the study, it was concluded that coronavirus infection can have a negative impact on the male reproductive system. The practical value of this study lies in improving approaches to medical care for men who have recovered from COVID-19 and creating preventive programmes.

## INTRODUCTION

In December 2019, scientists faced a new virological challenge when a new severe acute respiratory syndrome coronavirus 2 (SARS-CoV-2) was identified in China. According to data collected until November 2020, the coronavirus pandemic affected 188 countries and territories, with more than 55 million confirmed cases of the disease and over a million deaths caused by COVID-19 (Leng *et al*., 2023). The World Health Organization (WHO) classified this situation as a pandemic on March 11, 2020. The SARS-CoV-2 pandemic put incredible pressure on medical systems worldwide and caused significant social and economic changes. These consequences affect all areas of public life, including education, employment, mental health, and social stability (Chen *et al*., 2021).

Nevertheless, scientists still primarily focus on the direct impact of the new coronavirus infection on health. This disease poses a serious threat due to its potential lethality and possible long-term consequences. Preliminary studies indicate a greater susceptibility of men to COVID-19 compared to women. It is noted that men with COVID-19 more frequently develop complications, and their mortality rate is higher (Livingston & Bucher, 2020). This issue raises questions about the impact of COVID-19 on different population groups and the need to consider these differences when developing treatment and prevention strategies. In particular, it is critically important to understand how COVID-19 affects men’s reproductive health. It is necessary to investigate the effect of COVID-19 on the structure and function of male reproductive organs, including biological studies aimed at understanding the mechanisms through which SARS-CoV-2 affects the male reproductive system and epidemiological studies to determine the overall risk level for the male population.

Such studies can have serious implications for public health, as reproductive health affects not only the health of individual men but also that of their partners, offspring, and future generations (Zhang *et al*., 2020). Researchers from the West Kazakhstan Marat Ospanov Medical University, Urazaeva *et al*. (2022), made a significant contribution to the discussion of this subject. They noted that in 29 high-income countries, about one million additional deaths were registered in 2020, and in almost all countries, the excess mortality rate was higher for men than for women. In 2022, gender inequality in mortality further increased in most countries. In many of them, excess mortality significantly exceeded the registered deaths from COVID-19.

Although the coronavirus primarily causes respiratory symptoms, a significant portion of infected men is of reproductive age; thus, their fertility might be compromised. There is data indicating that viral diseases are often accompanied by hyperthermia, which affects sperm parameters (Livingston & Bucher, 2020). Ashilova *et al.* (2022) note that the virus can directly infect testicular cells, causing their damage and reducing spermatogenesis. This can lead to temporary or even permanent reduction in spermatogenesis and infertility in men and an increased risk of genetic disorders in future offspring. Therewith, Erkenova *et al.* (2022) state that COVID-19 can cause systemic inflammation and damage to blood vessels, including small blood vessels that supply blood to the genital organs. This can lead to impaired microcirculation and decreased tissue oxygenation, including the testicles. Insufficient blood supply and oxygenation can negatively impact the health and function of the testes.

Ibrayeva *et al*. (2022) concluded that one of the main problems related to COVID-19 and the male reproductive system is the virus’s impact on hormone levels. Studies show that patients with COVID-19 have increased levels of cytokines such as interleukin-6 (IL-6), which can lead to testicular dysfunction and worsen sperm quality. A decrease in testosterone levels, the main male sex hormone, is also noted, which can lead to decreased libido and erectile dysfunction. Furthermore, Ashilova *et al.* (2022) concluded that men with progressive chronic diseases such as diabetes, hypertension, and obesity have a higher risk of developing severe forms of COVID-19. These diseases can also negatively impact male reproductive health, exacerbating existing issues related to the virus.

The purpose of this study is to examine the impact of COVID-19 on the male reproductive system to determine the mechanisms and consequences of this impact, which will enable the development of effective prevention and treatment strategies and support male reproductive health during the pandemic and in the long term.

## MATERIALS AND METHODS

To address the set tasks and obtain necessary data for analysing the impact of COVID-19 on the male reproductive system, a range of appropriate methods was employed. The study utilised the meta-analysis method of medical organisation databases, the representative sample method, and the quantitative survey method. To obtain statistical information about the impact of COVID-19 on the male reproductive system, a complex set of procedures was employed. One of the key components chosen was the universal meta-analysis method of the international medical organisation database, including WHO, Global Health 50/50 (2023). Using this method, all available publications on the subject were searched and analysed. The results of studies conducted in major centres, hospitals, and universities were also included in the analysis. Statistical data of observations were obtained, which were used to assess the scale of the problems and analyse situations in various regions worldwide. The male-to-female mortality ratio was calculated using the formula (1):


(1) $$\text{ Case fatality rate }\left(\frac{m}{f}\right)=\frac{\text{ Number of deaths }(m/f)}{\text{ Number of cases }(m/f)}*100\text{, }$$

## where: *m* = males; *f* = females.

The mortality rate (men) is also calculated according to the formula (2):


(2) $$\text{ Case fatality rate }(m)=\frac{\text{ Case fatality rate }(m)}{\text{ Case fatality rate }(f)}*100,$$

## where: *m* = males; *f* = females.

Furthermore, this method allowed establishing a connection between COVID-19 and male reproductive functions and identifying factors that might be important in examining the reproductive health of the male population. The representative sample method helped in forming a group of people with specific, non-random characteristics. This method involves identifying a group of participants and subsequently recruiting them from the population coverage. In this case, men who were infected with COVID-19 and of reproductive age (typically defined as ages 18 to 55) were identified as the group for inclusion in the sample. This approach enables studying a specific subgroup of the population, providing valuable data and necessary information about the virus’s impact on the male reproductive system, identifying risk factors, and assessing the effectiveness of measures to restore reproductive health. This is a crucial step in the development and management of men’s health during increased COVID-19 pandemic.

As part of the study, a mass survey of a representative sample was conducted in the Republic of Kazakhstan using a well-developed sampling and a pre-created questionnaire. Through the quantitative survey method, 213 men aged 18-55 from different regions of the Republic of Kazakhstan were surveyed in 2023. The survey was conducted on the Internet service “SurveyPlanet”. An important aspect of the survey was ensuring the confidentiality and anonymity of the participants, receiving open and honest answers from them. Participants were informed about the study, and their data would be processed anonymously and confidentially. The survey results were systematised and analysed to identify the main problems of reproductive health in men infected as a result of coronavirus infection. This method allows obtaining objective and representative results, serving as the basis for developing concepts and considerations for protecting male reproductive system health during the pandemic.

## RESULTS

Analytical findings from WHO indicate no significant differences in the susceptibility of SARS-CoV-2 infection between men and women. Overall, the virus’ impact on populations in most countries was similar. However, some regions noted a higher infection rate among men compared to women. Despite this information, existing data indicate differences in the severity of the disease and the mortality rate between men and women. For instance, in China, the infection rate between genders was comparable, but the mortality rate among men was 4.7%, whereas among women, it was 2.8%. Similarly, in Italy, the mortality rate among men was 16.6%, while among women, it was 9.1%. The “Global Health 50/50 (2023) initiative provided data on SARS-CoV-2 infection and mortality rates broken down by gender for countries significantly affected by the pandemic. Some countries, including Brazil, India, the USA, and the UK, did not provide information on the gender composition of the infected individuals. To assess differences in mortality rates between men and women, the male-to-female mortality ratio was calculated based on the case fatality rate, using data provided by Global Health 50/50 (2023) (Table 1).

**Table 1:** Gender-specific data on COVID-19 cases and mortality for heavily affected countries.

Country	Number of cases		Number of deaths		η lethality		η mortality rate (m:f)
	(m)	(f)	(m)	(f)	(m)	(f)	
Thailand	1731	1417	44	14	2.54	0.98	2.59
Costa Rica	1275	1002	7	2	0.52	0.22	2.35
Albania	962	1085	20	10	2.08	0.91	2.28
Netherlands	18636	30995	3354	2741	17.99	8.84	2.03
Bosnia and Herzegovina	777	899	29	18	3.73	2	1.86
Haiti	3101	2110	48	17	1.54	0.8	1.92
North Macedonia	1641	1722	166	93	10.11	5.4	1.87
Denmark	5343	7218	342	261	6.4	3.61	1.77
Dominican Republic	14796	12574	456	213	3.08	1.69	1.81
England	98405	130337	21517	16147	21.86	12.38	1.76
Greece	1691	1394	130	60	7.68	4.3	1.78
Latvia	579	532	13	7	2.31	1.24	1.86
Northern Ireland	1850	3011	285	260	15.4	8.63	1.78
Romania	9863	12297	842	585	8.53	4.75	1.79
Spain	106759	141576	11629	8943	10.89	6.31	1.72
Sweden	24718	36120	2826	2335	11.43	6.46	1.76
Belgium	22470	38097	3543	3473	15.76	9.11	1.72
China	28577	27347	1343	771	4.69	2.81	1.66
Italy	109003	129047	19344	13865	17.74	10.74	1.65
Peru	149834	107613	5825	2398	3.88	2.22	1.74
Burkina Faso	599	335	28	10	4.72	2.89	1.63
Ecuador	16448	13167	2214	1120	13.46	8.5	1.58
Mexico	101873	83249	14926	7658	14.65	9.19	1.59
Scotland	6853	11185	2051	2068	29.92	18.48	1.61
Switzerland	14348	16958	967	713	6.73	4.2	1.6
Ukraine	4140	5270	132	107	3.18	2.03	1.57
Afghanistan	18846	7141	336	87	1.78	1.21	1.46
South Korea	5314	7170	150	131	2.82	1.82	1.54
Bangladesh	82208	33578	1157	345	1.4	1.02	1.36
Czech Republic	5113	5069	197	142	3.85	2.8	1.37
Indonesia	25241	22655	1536	999	6.08	4.4	1.38
Kenya	3269	1469	93	30	2.85	2	1.42
South Africa	45177	60131	1087	1013	2.4	1.68	1.42

*Note: m - males; f - females; η - coefficient.*

*Source:* (Global Health 50/50. 2023).

Analysis of data from 33 countries showed that the mortality rate fluctuated between 2.8 and 1.4, as presented in Table 1. The results indicate a high mortality rate from COVID-19 among men and women in the specified countries. A higher mortality coefficient among men suggests a possible increased vulnerability of the male population to disease forms. However, it is essential to consider that these results might be associated with other various factors, including age, comorbidities, and socio-economic conditions, which can differ in different countries. Recent studies conducted by the Kazakh National Medical University have focused on examining the impact of coronavirus infection on men’s reproductive health. Based on the research results, scientists found that the SARS-CoV-2 virus, by binding to the angiotensin-converting enzyme 2 (ACE2) receptor, causes significant changes in the regulation of autophagy in Leydig and Sertoli cells, and in the seminiferous tubules. These changes play a crucial role in sperm production and accumulation. This, in turn, can negatively affect sperm quality and a man’s ability to conceive successfully. To obtain more precise information on the impact of COVID-19 on sperm parameters, data on characteristics before and after infection were included (Table 2).

**Table 2:** Semen parameters of participants before COVID-19 and after COVID-19.

Number of patients	Volume (ml)		Sperm concentration (million/ml)		Progressive motility (%)		Total mobility (%)	
	Before	After	Before	After	Before	After	Before	After
21 (s.s.)	3±1.3	2.5±1.2	42±20	35.9±20.1	35.1±21.7	21.8±15.9	48.60±22.1	34.7±20.7
41 (s.s.)	2.61±1.25	2.25±1.03	24±17	13±18	33.66±13.59	30.74±14.28	42±15.52	38.7±16.35
24 (s.s.)	3.6±1.6	3.30±1.5	42.6±18	35.3±20.2	34.5±1.5	28.9±9.1	45.8±5	40.4±10.9
26 (l.s.)	3.24±1.6	3.18±0.8	32.24±12.8	30.62±12.4	28.81±9.7	20.92±9.1	48.69±12.1	33.41±12.3
43 (m.s.)	3.34±1.1	2.74±0.9	35.01±14.1	30.63±17.2	30.16±12.1	21.40±10.1	50.74±13.4	31.42±13.3
29 (s.s.)	2.53±1.11	2.08±1.01	41.67±40.45	36.52±60.84	32.62±12.59	26.24±15.49	40.23±15.06	33.97±19.07

*Note: s.s. - severe symptoms; m.s. - moderate symptoms; l.s. - light symptoms.*

*Source: compiled by the authors.*

**Figure 1:**
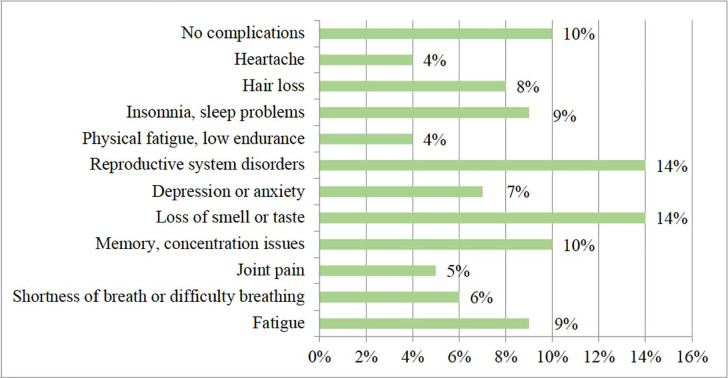
Complications arising after COVID-19 disease in the male population of the Republic of Kazakhstan. *Source: compiled by the authors*.

The study revealed that most patients infected with COVID-19 experienced decreased total sperm motility. In addition, progressive motility, sperm concentration, and volume also significantly decreased in some semen samples. This reduction in sperm parameters likely correlates with the severity of the disease. Specifically, all measured sperm parameters significantly decreased in patients with moderate and severe symptoms of COVID-19, while patients with mild symptoms experienced a reduction only in progressive motility and total motility. These results indicate the potential negative impact of the virus on male reproductive function, which may have long-term consequences for fertility and reproductive health. Further research is necessary to fully understand the mechanisms and consequences of SARS-CoV-2’s impact on male reproduction. Moreover, other factors such as patients’ age and the presence of comorbidities should be considered to determine the link between COVID-19 and male reproductive function more accurately. To conduct a more extensive and detailed study of the consequences of COVID-19 on the male reproductive system, additional research was conducted among men of reproductive age who were infected with the SARS-CoV-2 virus in the Republic of Kazakhstan. These studies included a survey in which 213 respondents from various regions of the country participated and aimed to identify potential complications associated with COVID-19 (Figure 1).

**Figure 2:**
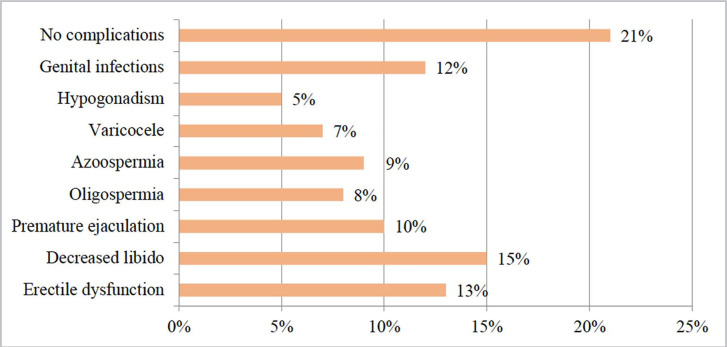
Complications in the reproductive system of the male population of the Republic of Kazakhstan that arose after the COVID-19 disease. *Source: compiled by the authors*.

The survey results identified several complications that arose after recovering from COVID-19. In the analysis of the data presented in Figure 1, it is noteworthy that despite most cases of the new coronavirus infection (COVID-19) being characterised by mild complications such as loss of smell or taste (14%) and memory and concentration problems (10%), significant issues also arise in the reproductive system (14%). This fact necessitates further assessment of potential risks of COVID-19’s impact on the male reproductive system. These results highlight the need for further research and understanding of the influence of COVID-19 on the male reproductive system. Such risk assessment will aid in developing effective prevention and treatment measures and providing relevant information and support to men dealing with the consequences of the disease. Subsequent surveys revealed specific symptoms indicating disruptions in the male reproductive system after recovering from COVID-19 (Figure 2).

The study found that only 21% of men who had recovered from COVID-19 did not experience complications in the reproductive system. Among the list of complications, the most common responses were decreased libido (15%), impotence (13%), and genital infections (12%). The obtained results point to the potential impact of COVID-19 on the functioning of the male reproductive system. These disruptions in reproductive system function are serious issues that can significantly affect men’s quality of life and fertility. To assess disruptions in the field of men’s reproductive health, respondents were asked the following additional yes-or-no questions (Table 3).

**Table 3:** Additional questions about reproductive health problems among the male population of the Republic of Kazakhstan.

Question	Yes	No
Have you had infections of the genital organs or inflammatory diseases of the reproductive system before getting infected with COVID-19?	14%	86%
Do you consume substances such as alcohol or drugs that may have negative consequences for reproductive health?	33%	67%
Have you or your family members experienced cases of hypogonadism, genetic, or congenital defects of the reproductive system?	9%	91%
Have you undergone surgeries or observations related to the reproductive system?	6%	94%
Are you taking any medications that affect reproductive health?	25%	75%
Do you believe that the stress caused by COVID-19 can affect reproductive health?	84%	16%
Do you frequently experience stress in your daily life?	66%	34%
Have you had consultations or treatment related to reproductive health disorders?	21%	79%
Do you have chronic diseases such as diabetes, hypertension, or cardiovascular diseases?	25%	75%
Are you aware of the availability of medical services or programmes related to men’s reproductive health?	6%	94%

*Source: compiled by the authors*.

According to the survey results, most surveyed men from the Republic of Kazakhstan are aware of the impact of stress caused by COVID-19 on reproductive health. However, a smaller portion of them has experience or awareness of other aspects of male reproductive health, such as infections, disease onset, genetic defects, chronic conditions, and available medical services. This lack of awareness might be due to insufficient public knowledge about male reproductive health, possibly stemming from the absence of scientific programmes and informational resources dedicated to this subject. The results obtained provide for broader information and preventive measures in the field of male reproductive health.

## DISCUSSION

The study results confirm the presence of issues in the reproductive health of men who have recovered from COVID-19. Moreover, the data also indicate a higher predisposition of male patients to mortality from COVID-19 and other acute viral infections compared to females. This is an important area for research and the development of measures for the prevention and treatment of these problems. Understanding the scale and characteristics of the impact of COVID-19 on the male reproductive system is part of the strategy for developing effective treatment methods and support for this vulnerable patient group. The discovery of the features associated with the emergence of the virus on male reproduction may manifest itself in the development of natural and biological therapeutic approaches (Malki, 2022).

A study by Mali *et al*. (2021) found that COVID-19 can cause testicular dysfunction in infected men. It was noted that the main receptor for SARS-CoV or SARS-CoV-2 became angiotensin-converting enzyme 2 (ACE2) receptor, primarily located in the heart, lungs, kidneys, and testes. This explains the adverse effects on the organs of the male reproductive system. Their study confirmed the observation that the infection can induce changes in spermatogenesis and sperm quality. This suggests that disruption in the male reproductive system is a direct manifestation of the virus’s action on the body. Therefore, according to the authors, identifying and minimizing the risk of testicular infection by the virus is one of the most important tasks, as the continuation of the lineage depends on it.

A recent study by Ghosh *et al*. (2022) thoroughly examined the impact of COVID-19 on the properties of seminal vesicles and ejaculates. The results showed an increased frequency of reproductive system disorders, as they found that patients infected with COVID-19 may experience serious dysfunction of seminal vesicles, leading to changes in ejaculate composition. Rapid changes in seminal fluid in infected men cause allergic reactions. Thus, these findings also confirm the observations made in the study that the onset of COVID-19 on the male reproductive system can have serious consequences and lead to noticeable changes in the ejaculation process and complete ejaculation.

The study conducted by Xie *et al*. (2022) suggests that the COVID-19 virus can affect reproductive tissues. In their work, the authors report the pronounced expression of ACE2 receptors in both male and female reproductive organs. This plays a crucial role in sperm function and egg fertilisation. The authors also note the increased expression of ACE2 receptors in testicular tissues, especially in spermatogonia (spermatozoon-producing cells), Sertoli cells (cells supporting spermatogenesis), and Leydig cells (cells synthesising testosterone), and in clusters of epithelial cells of the prostate gland, fibroblasts, and pericytes. Lower expression of ACE2 is observed in spermatocytes (mature spermatids), late spermatocytes, and somatic cells. In addition, there is high activity of the protease TMPRSS2 in embryonic and somatic cells. It is worth noting that male sex hormones (androgens) act as regulators of TMPRSS2 gene transcription. This is due to an androgen-responsive element in the TMPRSS2 gene, consisting of 15 base pairs (bp). These findings are associated with the possible influence of the COVID-19 virus on reproductive tissues and the emergence of corresponding reproductive system disorders, such as oligozoospermia, azoospermia, and varicocele. Similar disruptions in the reproductive system were observed in a portion of the male population of the Republic of Kazakhstan.

Research results show that COVID-19, caused by the SARS-CoV-2 virus, can lead to complications such as insomnia, cough, loss of smell, or taste. However, over time, it was observed that patients with COVID-19 may experience negative effects on various organs and systems in the body, including the reproductive system. Patients recovering from COVID-19 report some temporary issues with erection and sexual desire. Various disturbances have been noted in some men who have recovered from COVID-19, including reduced sperm quality, changes in hormonal balance, increased risk of erectile dysfunction, and premature ejaculation. In some cases, inflammatory processes in the genital organs were also registered. Men’s reproductive health plays a crucial role in societal well-being and family planning, the study of which becomes a part of broad medical science. Understanding these mechanisms operating within the framework of individual protection can aid in implementing prevention and treatment strategies. Based on the above, global research on this issue can help respond proactively to future outbreaks and develop an international strategy for protecting and preventing cases of reproductive disorders in men (Hamarat *et al*., 2022).

Several studies have been conducted in different countries regarding the impact of COVID-19 on the male reproductive system. For instance, a study by Li *et al*. (2022) in China showed significant disruptions in male reproductive function in male patients recovering from COVID-19. These disruptions were marked by overproduction of seminal markers of inflammation and oxidative stress and activation of apoptotic variables. These changes persisted over time and inversely correlated with sperm quality parameters. The authors also demonstrated that COVID-19 can activate ACE2 in seminal plasma, suggesting that the semen of patients should be considered a vulnerable route for COVID-19 infection. It is noted that monitoring the reproductive functions of men recovering from the disease is essential, as they may develop transient male insufficiency, similar to patients with oligoasthenoteratozoospermia, where infected men experience decreased androgen (testosterone) levels and disturbed spermatogenesis.

Meanwhile, colleagues from Italy, Scroppo *et al*. (2021), observed changes in seminal fluid in almost all patients, both in microscopic and macroscopic characteristics, such as hypoposia and increased viscosity, which were not detected in previous studies. Sperm analysis, considering sperm concentration, progressive motility, and morphology, revealed changes in 13 out of 15 patients (87%); specifically, progressive motility was reduced in 11 patients (73%). If semen volume is added as a parameter, hypospermia was found in four patients (27%). These results confirm the observations and the international significance of covering COVID-19 in the male reproductive system.

Examining the male reproductive system in the context of COVID-19 helps establish a connection between the infection and potential issues with fertility, sexual activity, and other aspects of male health. Understanding the virus’s impact on these systems aids in developing prevention and treatment methods (Agrawal *et al*., 2021). A problem arises concerning the insufficient awareness of the population regarding men’s reproductive health and the lack of scientific programs and informational resources dedicated to this issue. This is supported by the survey results of the male population of the Republic of Kazakhstan, where the majority of respondents are unaware of the existence of medical services or programs related to men’s reproductive health (94%). The lack of comprehensive information and guidelines on male reproductive health can lead to misconceptions and misunderstandings about the relationship between COVID-19 and the male reproductive system (Zeginiadou *et al*., 2023).

Obtaining results necessitates the implementation of larger-scale informational and preventive measures in the field of men’s reproductive health. This includes developing and disseminating informational materials, conducting research, and involving healthcare professionals and organisations in disseminating and guiding this sphere (Tur-Kaspa *et al*., 2021). Scientific programmes and informational resources can serve as a source for creating informative and effective guidelines for preventing and reducing instances of disruptions in men’s reproductive health during the pandemic. They can offer recommendations on protection against the impact of COVID-19, risks, and potential effects on the reproductive system, as well as approaches to dealing with symptoms post-infection (Săndulescu *et al*., 2022). Consequently, the development and accessibility of scientific programmes and informational resources dedicated to men’s reproductive system during COVID-19 are critically important for educating the public, improving men’s health, and making informed decisions regarding health protection.

## CONCLUSIONS

The study confirmed that COVID-19 can impact the male reproductive system. Despite no significant increase in infection rates between men and women, differences in mortality and disease severity were observed among genders. Special attention was given to the elevated mortality rate among men, which is less common among women, emphasising the possible higher vulnerability of the male population. It was established that COVID-19 can negatively affect sperm parameters, including sperm concentration, progressive motility, total motility, and volume. Changes in these parameters were detected in most patients infected with the coronavirus, especially those experiencing moderate to severe symptoms. This could lead to a potential adverse effect of the virus on male reproductive function.

A survey was conducted among men of reproductive age in Kazakhstan, revealing several complications arising after recovering from COVID-19. The study identified possible issues: impaired concentration and memory (10%), insomnia (9%), loss of smell or taste (14%), and problems with the reproductive system (14%). Within serious complications, the reproductive system exhibited the most common symptoms, including decreased libido (15%), impotence (13%), and genital infections (12%). This study aimed to explore complications from COVID-19 and its impact on the male reproductive system. It is important to consider factors such as patients’ age and underlying health conditions for a more precise understanding of the relationship between COVID-19 and male reproductive systems. The survey also indicated that most men in Kazakhstan are aware of the impact of COVID-19-related stress on reproductive health. However, they have limited knowledge about other aspects of male reproductive health.

The absence of scientific programmes and informational resources necessitates economic and preventive measures in the field of male reproductive health. The study results highlight the potential threat of COVID-19 to the male reproductive system and suggest the implementation of broader informational, preventive programmes, and research in the area of male reproductive health to identify the consequences of COVID-19, its treatment, and support for the male population.

## References

[r1] Agrawal H, Das N, Nathani S, Saha S, Saini S, Kakar SS, Roy P. (2021). An Assessment on Impact of COVID-19 Infection in a Gender
Specific Manner. Stem Cell Rev Rep.

[r2] Ashilova MS, Begalinov AS, Begalinova KK. (2022). Transformation of the educational values of youth during the
COVID-19 pandemic. Prof Educ Mod World.

[r3] Chen F, Zhu S, Dai Z, Hao L, Luan C, Guo Q, Meng C, Zhang Y. (2021). Effects of COVID-19 and mRNA vaccines on human
fertility. Hum Reprod.

[r4] Erkenova SE, Lokshin VN, Saduakassova ShM, Dzhardemalieva NZh, Tazhekova AB, Jarikova BN, Abden AG, Zhumabek AK (2022). Impact of SARS-CoV-2 infection on the reproductive system
(literature review). Bull Asfendiyarov Kazakh Natl Med Univ.

[r5] Ghosh S, Parikh S, Nissa MU, Acharjee A, Singh A, Patwa D, Makwana P, Athalye A, Barpanda A, Laloraya M, Srivastava S, Parikh F. (2022). Semen Proteomics of COVID-19 Convalescent Men Reveals Disruption
of Key Biological Pathways Relevant to Male Reproductive
Function. ACS Omega.

[r6] Global Health 50/50 (2023). https://globalhealth5050.org/the-sex-gender-and-covid-19-project/.

[r7] Hamarat MB, Ozkent MS, Yilmaz B, Aksanyar SY, Karabacak K. (2022). Effect of SARS-CoV-2 infection on semen
parameters. Can Urol Assoc J.

[r8] Ibrayeva LK, Rybalkina DK, Minbayeva LS, Minbayev SK, Bacheva IV, Zharylgapova AM. (2022). Development of the COVID-2019 pandemic in
Kazakhstan. Med News North Caucasus.

[r9] Leng T, Guo Z, Sang Z, Xin Q, Chen F. (2023). Effect of COVID-19 on sperm parameters: pathologic alterations
and underlying mechanisms. J Assist Reprod Genet.

[r10] Li X, Lu H, Li F, Zhang Q, Wang T, Qiang L, Yang Q. (2022). Impacts of COVID-19 and SARS-CoV-2 on male reproductive function:
a systematic review and meta-analysis protocol. BMJ Open.

[r11] Livingston E, Bucher K. (2020). Coronavirus Disease 2019 (COVID-19) in Italy. JAMA.

[r12] Mali AS, Magdum M, Novotny J. (2021). COVID-19 impact on reproduction and fertility. JBRA Assist Reprod.

[r13] Malki MI. (2022). COVID-19 and male infertility: An overview of the
disease. Medicine (Baltimore).

[r14] Săndulescu MS, Văduva CC, Siminel MA, Dijmărescu AL, Vrabie SC, Camen IV, Tache DE, Neamţu SD, Nagy RD, Carp-Velişcu A, Manolea MM. (2022). Impact of COVID-19 on fertility and assisted reproductive
technology (ART): a systematic review. Rom J Morphol Embryol.

[r15] Scroppo FI, Costantini E, Zucchi A, Illiano E, Trama F, Brancorsini S, Crocetto F, Gismondo MR, Dehò F, Mercuriali A, Bartoletti R, Gaeta F. (2021). COVID-19 disease in clinical setting: impact on gonadal function,
transmission risk, and sperm quality in young males. J Basic Clin Physiol Pharmacol.

[r16] Tur-Kaspa I, Tur-Kaspa T, Hildebrand G, Cohen D. (2021). COVID-19 may affect male fertility but is not sexually
transmitted: a systematic review. F S Rev.

[r17] Urazaeva ST, Sharatdinova AS, Begalin TB, Tussupkalyeva KSh, Amanshieva AA, Urazaeva AB, Izimova R, Amanzhanova AA (2022). Еxcess mortality in the context of the COVID-19 pandemic
(literature review). Bull Asfendiyarov Kazakh Natl Med Univ.

[r18] Xie Y, Mirzaei M, Kahrizi MS, Shabestari AM, Riahi SM, Farsimadan M, Roviello G. (2022). SARS-CoV-2 effects on sperm parameters: a meta-analysis
study. J Assist Reprod Genet.

[r19] Zeginiadou T, Symeonidis EN, Symeonidis A, Vakalopoulos I. (2023). SARS-CoV-2 infection (COVID-19) and male fertility: Something we
should be worried about?. Urologia.

[r20] Zhang Y, Geng X, Tan Y, Li Q, Xu C, Xu J, Hao L, Zeng Z, Luo X, Liu F, Wang H. (2020). New understanding of the damage of SARS-CoV-2 infection outside
the respiratory system. Biomed Pharmacother.

